# Stealth MRSA: a diagnostic pitfall in invasive *Staphylococcus aureus* infection

**DOI:** 10.1128/asmcr.00030-26

**Published:** 2026-05-27

**Authors:** Tess A. Karre, Rudolf J. Kotula, Richard V. Goering

**Affiliations:** 1 Department of Pathology, Microbiology, and Immunology, University of Nebraska Medical Center 12284, Omaha, Nebraska, USA; 2 Department of Infectious Diseases, Nebraska Methodist Health System 6217 , Omaha, Nebraska, USA; 3 Department of Medical Microbiology and Immunology, Creighton University 6216, Omaha, Nebraska, USA; Pattern Bioscience, Austin, Texas, USA

**Keywords:** invasive *Staphylococcus aureus* infection, genotype-phenotype discordance, *mecA*-mediated resistance, stealth MRSA

## Abstract

**Background:**

Methicillin-resistant *Staphylococcus aureus* (MRSA) is commonly identified by phenotypic antimicrobial susceptibility testing or detection of the *mecA* gene encoding penicillin-binding protein 2a (PBP2a). A subset of isolates, referred to as oxacillin-susceptible MRSA (OS-MRSA) or “stealth MRSA,” demonstrates phenotypic susceptibility to oxacillin despite harboring *mecA*, creating a diagnostic and therapeutic challenge.

**Case Summary:**

We describe a case of invasive infection caused by a USA300 OS-MRSA strain in a patient with a soft tissue abscess and infective endocarditis. Repeated phenotypic testing demonstrated oxacillin susceptibility, while molecular assays detected the *mecA* gene. Additional phenotypic and genotypic analyses confirmed OS-MRSA. The patient was successfully treated with MRSA-active antimicrobial therapy following recognition of the genotype–phenotype discrepancy.

**Conclusion:**

This case demonstrates the limitations of phenotypic susceptibility testing alone for detection of methicillin resistance in *S. aureus*. Supplemental testing beyond routine phenotypic susceptibility may be needed for accurate classification and appropriate management of OS-MRSA, particularly in invasive infections where failure to recognize resistance may lead to suboptimal therapy.

## INTRODUCTION

Methicillin-resistant *Staphylococcus aureus* (MRSA) is typically identified by phenotypic antimicrobial susceptibility testing or detection of the *mecA* gene encoding penicillin-binding protein 2a (PBP2a). However, oxacillin-susceptible *mecA*-positive isolates, commonly referred to as oxacillin-susceptible MRSA (OS-MRSA) or “stealth MRSA,” have been increasingly recognized and may evade routine laboratory detection, posing a diagnostic challenge (1). Variable expression of methicillin resistance and heteroresistance further complicates phenotypic testing in these isolates (2, 3).

## CASE PRESENTATION

A 47-year-old man with no prior chronic illnesses presented to the hospital with confusion and weakness. Approximately 1 week prior to presentation, the patient experienced blunt force trauma to the left chest wall and subsequently developed pain and swelling in the left chest wall and axillary region with a fluid collection suggestive of abscess formation. Laboratory evaluation revealed a blood glucose level of 1,136 mg/dL (70–140 mg/dL), an elevated anion gap of 28 mmol/L (4–12 mmol/L), and a pH of 7.31 (7.35–7.45), consistent with a new diagnosis of diabetes mellitus with diabetic ketoacidosis. Drainage of the chest wall fluid collection was submitted for culture, and this grew *Staphylococcus aureus*, which tested as susceptible to oxacillin with an MIC of 0.5 μg/mL by automated antimicrobial susceptibility testing (AST) utilizing the Phoenix system, which was interpreted as susceptible per Clinical Laboratory Standards Institute (CLSI) breakpoints. Based on these AST results, this isolate was reported as methicillin-susceptible *S. aureus* (MSSA). Blood cultures collected in the emergency department flagged as positive, and Gram’s stain demonstrated gram-positive cocci in clusters. A rapid molecular blood culture ID using the Verigene System (Luminex, Austin, TX, USA) was positive for the *S. aureus* and *mecA* targets. A transesophageal echocardiogram showed possible vegetation on the mitral valve chordae tendineae. Therefore, the patient was treated for bacterial endocarditis. Other than the chest wall abscess and likely infective endocarditis, no other metastatic foci of infection were identified upon imaging.

Susceptibility testing of the bloodstream isolate performed on the Phoenix Automated AST System (Becton Dickinson, Franklin Lakes, NJ, USA) showed an oxacillin MIC of 0.5 μg/mL, which was unexpected given the presence of *mecA* by molecular testing. Multiple subsequent blood cultures grew *S. aureus* with the same genotype–phenotype discrepancy.

Upon recognition of the discrepancy, additional genotypic and phenotypic studies were performed on the bloodstream and soft tissue isolates. Testing by disk diffusion following exposure to beta-lactam antibiotics using techniques described previously (3) to assess inducible or unstable methicillin resistance demonstrated a shift to methicillin resistance. Genotypic characterization by pulsed field gel electrophoresis (PFGE) (4) and whole-genome sequencing (WGS) (3) confirmed that the chest wall and bloodstream isolates were genetically indistinguishable from the USA300 (ST8, SCC*mec*IVa) strain type and represented oxacillin-susceptible MRSA (OS-MRSA), sometimes referred to as “stealth MRSA.” An immunochromatographic membrane assay detected penicillin-binding protein 2a (PBP2a) in both isolates.

The phenotypic oxacillin susceptibility observed in the chest wall isolate, if considered in isolation, would have supported treatment as MSSA. Because, in this case, the blood cultures obtained at presentation became positive shortly thereafter, and rapid molecular testing of the bloodstream isolate detected the presence of *mecA* at approximately the same time as abscess culture susceptibilities, the patient was not de-escalated to MSSA-directed beta-lactam therapy.

The patient was treated with 7 days of intravenous daptomycin (700 mg q 24 h) and ceftaroline (600 mg q 12 h) due to persistent positive cultures. This was followed by 35 days of daptomycin monotherapy after clearance of bacteremia, with sustained clinical improvement. Because diabetes and hyperglycemia are known risk factors for severe bacterial disease, management of hyperglycemia and diabetic ketoacidosis was a critical adjunct to antimicrobial therapy.

## DISCUSSION

Methicillin-resistant *S. aureus* (MRSA) is a pathogen of concern due to its ability to cause serious infections resistant to β-lactam antibiotics, such as methicillin and oxacillin. Because *S. aureus* colonizes the skin and mucocutaneous surfaces, it spreads in both healthcare and community settings. MRSA most frequently causes skin and soft tissue infections, bacteremia, endocarditis, and osteoarticular infections (5).

Methicillin resistance in *S. aureus* is mediated by acquisition of the *mecA* gene typically located on the staphylococcal cassette chromosome mec. The *mecA* gene encodes penicillin-binding protein 2a (PBP2a), which has reduced affinity for β-lactam antibiotics and allows cell wall synthesis to continue in the presence of these agents. Laboratory techniques to identify MRSA include both phenotypic (broth microdilution AST, disk diffusion, PBP2a latex agglutination or immunochromatographic assay) and genotypic methods, such as PCR. Although most MRSA isolates are readily identified using standard laboratory workflows, the emergence of isolates with discordant genotypic and phenotypic results, commonly referred to as oxacillin-susceptible MRSA (OS-MRSA) or “stealth MRSA,” has posed diagnostic challenges for clinical laboratories. These may test susceptible to oxacillin and, in some cases, fail to demonstrate resistance to cefoxitin despite the presence of *mecA* (2). Exposure to subinhibitory concentrations of beta-lactam antibiotics can induce *mecA*-mediated resistance in such isolates (6).

In this case, had molecular testing of the bloodstream isolate not been performed or had blood culture results been delayed, the oxacillin-susceptible phenotype of the abscess isolate could have reasonably prompted treatment with standard MSSA-directed agents, such as cefazolin, nafcillin, or oxacillin. Such therapy would not provide reliable coverage for *mecA*-positive organisms and may permit re-emergence of beta-lactam resistance under antibiotic pressure. Recognition of OS-MRSA, therefore, directly impacts antimicrobial selection.

In some OS-MRSA isolates, genotype–phenotype discordance appears to result from sequence instability of *mecA*. Goering et al. identified mutations in *mecA* nucleotide repeat regions in clinical OS-MRSA isolates that were oxacillin-susceptible and PBP2a-negative, suggesting that molecular detection of *mecA* was required for their identification (3).

In contrast to previously described OS-MRSA isolates that were oxacillin-susceptible and PBP2a-negative due to *mecA* sequence instability, both isolates in this case were PBP2a-positive by immunochromatographic membrane assay. This finding confirms the presence and expression of *mecA* at the protein level despite phenotypic susceptibility to oxacillin supporting the concept that OS-MRSA may arise from mechanisms other than complete loss or silencing of PBP2a. Tao et al. described PBP2a-positive isolates that remained susceptible to oxacillin and cefoxitin, implicating functional differences in PBP2a rather than complete loss or silencing of *mecA* expression (6). Functional characterization of PBP2a was not performed, and WGS analysis revealed no mutations within the *mecA* gene. However, missense mutations were discovered in clpX (arg259ser) and clpL (ser249cys). While not explored further, *clp* genes have been associated with *mecA* expression (7), underscoring the growing recognition that diverse underlying mechanisms contribute to genotype–phenotype discordance in OS-MRSA.

Prior studies have demonstrated phenotypic heterogeneity in OS-MRSA, with some oxacillin-susceptible, mecA-positive strains retaining cefoxitin resistance depending on the underlying mechanism (2, 3, 6). Because this was a retrospective case, definitive cefoxitin susceptibility data were not available for the isolate described here.

This case illustrates that USA300 can also present as OS-MRSA. Because USA300 predominates in community-associated MRSA infections and is associated with enhanced virulence mediated by mobile genetic elements and *agr*-regulated toxin expression, failure to recognize methicillin resistance in such isolates may result in inappropriate therapy and adverse outcomes (5).

Studies have reported the prevalence of OS-MRSA among clinical *S. aureus* isolates to be approximately 1 to 25%, with variability depending on patient population and geography (3). This variability has prompted consideration of whether supplemental testing beyond routine phenotypic susceptibility should be applied to detect *mecA*-mediated resistance. Molecular *mecA* testing and/or PBP2a testing has been proposed as an adjunct to standard susceptibility testing algorithms (3, 8). However, because some OS-MRSA isolates may be PBP2a-negative, molecular *mecA* testing may be needed in selected cases. The utility and cost-effectiveness of additional testing, whether molecular or PBP2a-based, are likely to depend on specimen type, local prevalence of OS-MRSA, and institutional testing practices.

Treatment for OS-MRSA should consider the presence of *mecA*, regardless of phenotypic susceptibility. Selection of antimicrobial therapy depends on the clinical syndrome and may include MRSA-active agents, such as vancomycin, daptomycin, or linezolid. Ceftaroline, a cephalosporin with activity against MRSA, may be used for selected infections, including skin and soft tissue infections and, in some cases, bloodstream infections when MRSA-active beta-lactam therapy is desired. Second-generation lipoglycopeptides, including dalbavancin, telavancin, and oritavancin, may be effective in appropriate clinical settings, such as uncomplicated skin and soft tissue infections or bacteremia in clinically stable patients, when prolonged parenteral therapy is required, and outpatient management is desirable, and when deep-seated infections, such as endocarditis, have been reasonably excluded.

Cefoxitin has stronger induction of *mecA* and improved sensitivity for PBP2a-mediated resistance (9). However, oxacillin-susceptible *mecA*-positive isolates may demonstrate susceptibility to both oxacillin and cefoxitin, highlighting the limitations of phenotypic testing alone in OS-MRSA (2).

This case underscores the clinical significance of genotype–phenotype discordance in *S. aureus* and the importance of supplemental testing to ensure accurate detection of methicillin resistance. CLSI guidelines state that detection of *mecA* or PBP2a represents the most definitive indicator of methicillin resistance in *S. aureus. S. aureus* isolates that test positive for *mecA* or PBP2a should be reported as methicillin resistant, regardless of phenotypic susceptibility results (9).
